# Selective Persistence of HPV Cross-Neutralising Antibodies following Reduced-Dose HPV Vaccine Schedules

**DOI:** 10.3390/vaccines7040200

**Published:** 2019-11-28

**Authors:** Zheng Quan Toh, Jennie Kosasih, Fiona M. Russell, Rita Reyburn, James Fong, Evelyn Tuivaga, Felisita T. Ratu, Cattram D. Nguyen, Silivia Matanitobua, Lien Anh Ha Do, Trevelyan Menheniott, Ian H. Frazer, Suzanne M. Garland, Edward Kim Mulholland, Paul V. Licciardi

**Affiliations:** 1 Infection and Immunity, Murdoch Children’s Research Institute, Parkville, VIC 3052, Australia; zheng.quantoh@mcri.edu.au (Z.Q.T.); ajenniekosasih@gmail.com (J.K.); fmruss@unimelb.edu.au (F.M.R.); rita.reyburn@gmail.com (R.R.); cattram.nguyen@mcri.edu.au (C.D.N.); lienanhha.do@mcri.edu.au (L.A.H.D.); trevelyan.menheniott@mcri.edu.au (T.M.); suzanne.garland@thewomens.org.au (S.M.G.); kim.mulholland@lshtm.ac.uk (E.K.M.); 2 Department of Paediatrics, The University of Melbourne, Parkville, VIC 3052, Australia; 3 Ministry of Health and Medical Services, Suva 679, Fiji; james.fong@health.gov.fj (J.F.); evelyntuivaga@gmail.com (E.T.); tupou.ratu@gmail.com (F.T.R.); siliviaus@yahoo.com (S.M.); 4 Diamantina Institute, The University of Queensland, Brisbane, QLS 4072, Australia; i.frazer@uq.edu.au; 5 Department of Obstetrics and Gynecology, University of Melbourne, Parkville, VIC 3052, Australia; 6 Regional WHO HPV Reference Laboratory, Centre Women’s Infectious Diseases Research, The Royal Women’s Hospital, Parkville, VIC 3052, Australia; 7 Department of Infectious Disease Epidemiology, London School of Hygiene and Tropical Medicine, London WC1E 7HT, UK; 8 Department of Child Health, Menzies School of Health Research, Darwin, NT 0811, Australia

**Keywords:** human papillomavirus vaccine, reduced doses, cross-neutralising antibodies, low- and middle-income countries

## Abstract

The duration of cross-neutralising antibody responses (cross-NAb) following HPV immunisation is unknown. We compared cross-NAb responses in cohort of girls who were either unimmunised or had received immunisation with one, two or three doses of 4vHPV (Gardasil^®^, Merck Inc., Kenilworth, NJ, USA) six years earlier, before and one month after a booster dose of 2vHPV (Cervarix^®^, GSK, Brentford, UK). NAb to potentially cross-reactive HPV genotypes 31, 33, 45, 52 and 58 were measured using a HPV pseudovirion-based neutralisation assay. Girls who had previously received at least one dose of 4vHPV had significantly higher NAb titres for HPV31 when compared with unimmunised girls, whereas no difference in NAb titre was observed for four other genotypes (33, 45, 52 and 58). Following a single further immunisation with 2vHPV, NAb titres to each of the five tested HPV genotypes were comparable for girls who previously received one, two or three doses of 4vHPV, and were significantly higher than for previously unimmunised girls. Immunisation with one, two or three doses of 4vHPV induced NAb to HPV31 that persisted for six years, but there was no persistence of NAb to HPV33, 45, 52 or 58. Our results suggest that one or two doses of 4vHPV may provide long-term protection against HPV31.

## 1. Introduction

Bivalent (HPV16, 18) (Cervarix^®^) and quadrivalent (HPV6, 11, 16, 18) (Gardasil^®^) human papillomavirus (HPV) vaccines, when administered in a standard three-dose schedule (0, 1 or 2, and 6 months) have demonstrated effective protection >96% against HPV type specific cervical cancer precursors in previously uninfected individuals [1,2,3,4,5]. Both vaccines also induce high levels of vaccine type-specific antibodies that persist for at least 12 years [6].

HPV16 and HPV18 are the commonest oncogenic HPV genotypes, while HPV31, 33, 45, 52 and 58 are the next most common, together accounting for 20% of cervical cancer cases worldwide [7]. These 5 genotypes are phylogenetically-related to HPV16 and 18. HPV 31, 33, 52 and 58 belong to the same Alpha 9 (A9) species group as HPV16, while HPV45 and HPV18 are in the Alpha 7 (A7) group [8] While previous studies have demonstrated immunisation efficacies of 20–60% against HPV infection and cervical cancer precursor lesions caused by HPV31/33/45/52/58 in women given three doses of 2vHPV or 4vHPV [9,10,11], a recent post-hoc analysis of Phase III clinical studies have revealed significantly higher efficacy against non-vaccine type high grade cervical lesions in 2HPV when compared with 4vHPV [12]. In countries that have introduced either 2vHPV or 4vHPV immunisation, effectiveness of 30–60% against HPV31, 33 and 45 associated disease have also been reported after five years, suggesting cross-protection [13,14,15]. This cross-protection is thought to be mediated by cross-neutralising antibodies (cross-NAb) to the L1 capsid protein that share homologous sequences within the A9 and A7 species group, although other mechanisms (e.g., cross-reactive T-cells) may also be important [12]. NAb to HPV31, 33 and 45 were generated in a proportion of individuals following three doses of 2vHPV or 4vHPV [16,17,18,19]. However, these cross-NAb are of approximately 100-fold lower titre than vaccine genotype specific antibodies [17,19], and it is not known how long these antibodies persist in the circulation.

Since 2015, two doses of HPV vaccine given six months apart has been recommended by the World Health Organization for girls under 15 years old [20]. Furthermore, there is promising observational data on the immunogenicity and efficacy of single-dose HPV vaccine schedules against vaccine-type HPV infection, and data from randomised controlled trials are anticipated [11,21,22,23]. The question of whether reduced-dose schedules generate similar durable NAb to potentially cross-reactive HPV genotypes 31, 33, 45, 52 and 58 as the three-dose schedule is unknown.

We performed a cohort study in Fiji to examine the immunogenicity of reduced-dose 4vHPV vaccine schedules in girls previously unimmunised or immunised with one, two or three doses of 4vHPV. The vaccine genotype-specific NAb and cellular immune response data have been published previously [22,24]. Here, we examined the NAb responses to HPV31, 33, 45, 52 and 58, six years after the last dose of 4vHPV and one month after a booster dose of 2vHPV.

## 2. Materials and Methods

### 2.1. Study Design, Procedures and Participants

The details of the study design, enrolled participants as well as study procedures have been described previously [22]. Briefly, a cohort study was conducted in Fiji between February and March 2015. A total of 200 girls aged 15–19 years who were previously immunised with one, two or three doses of 4vHPV, and girls who had not received any HPV vaccine were recruited for the study. Each dosage group were comprised of equal proportions of the two main ethnic groups—iTaukei (IT) and Fijians of Indian Descent (FID). Girls who had previously received one, two or three doses of 4vHPV were recruited through the school immunisation record obtained from the Fiji Ministry of Health and Medical Services, as well as friends of recruited girls, due to difficulties in locating girls on the vaccine list, while girls who had not received any prior HPV vaccine were recruited by the recommendation of friends of recruited girls and by informal network (i.e., word of mouth). A single dose of 2vHPV (Cervarix^®^, GlaxoSmithKline, Belgium) was given at day 0 to all participants to evaluate the immunological memory following reduced-dose schedules, and blood samples were collected to determine HPV specific immune responses prior to and 28 days following immunisation.

### 2.2. Laboratory Methods

The plasmids (p31sheLL, pVITRO-HPV33L1L2, p45sheLL, p52sheLL and p58sheLL) used to produce the pseudovirions were gifts from John Schiller and Richard Roden. The NAbs against HPV types 31, 33, 45, 52 and 58 were measured using the pseudovirion-based neutralisation assay previously described [18]. A neutralising titre (ED50) was defined as the highest serum dilution that reduces the secreted alkaline phosphatase activity by at least 50% in comparison to control (pseudovirions without serum). A sample with an ED50 value of ≥25 was considered HPV seropositive in our assay; seronegative samples were given a value of 12.5. All laboratory staff were blinded to the immunisation status of each participant, and each sample was identified according to a unique study number.

### 2.3. Statistical Analysis

The primary analysis was the comparison of the geometric mean titres (GMTs) of the HPV-specific NAb titres against HPV31, 33, 45, 52 and 58 in girls who previously received zero, one or two doses of 4vHPV with girls who received three doses. The secondary analyses were the comparison of the NAb GMTs one month post-2vHPV between girls who received zero, one or two doses and girls who received three doses. We also stratified the girls in each dosage group by ethnicity and compared their NAb GMTs based on our previous findings that vaccine-type NAb titres differed by ethnicity [22]. Within the two-dose group, we also stratified the girls into those who received two doses <6 months or ≥6 months apart, and compared their NAb GMTs before and one month after 2vHPV. For these analyses, we log-transformed the NAb titres and compared them using the Student’s *t* test or Mann–Whitney test (for comparison of NAb titres between iTaukei and FID within the zero-dose group only). The seropositivity rates were compared between the three-dose group and zero, one or two dose groups using the Fisher’s Exact test. Correlation analyses between vaccine-type GMTs (from [22]) and NAb GMTs to HPV31, 33, 45, 52 and 58 were performed using the Spearman’s correlation analyses. All statistical analyses were performed using GraphPad Prism software, version 5.0. For the primary outcome analysis, based on results from a two-year follow-up study [16], a sample size of 60 per group provided 84% power to detect a fold change in means (expected ratio) of 1.65 assuming that the coefficient of variation is 1.15 using a two group *t*-test with a 0.05 two-sided significance level.

### 2.4. Ethics Approval

The study was approved by the Fiji National Research Ethics Review Committee, Fiji National Research Committee (2014.5.FNRERC.5.SU), as well as the Royal Children’s Hospital Human Research Ethics Committee, Melbourne, Australia (34239A). The study was registered with clinicaltrials.gov, number NCT02276521.

## 3. Results

The baseline characteristics of the study participants were described in Toh et al. [22]. Briefly, a total of 200 girls were recruited: 66 (three-dose group); 60 (two-dose group); 40 (one-dose group); 34 (zero-dose group). Three girls (two from the zero-dose group and one girl from the two-dose group) were lost to follow-up for the post-immunisation visit, and two girls in the zero-dose group were excluded from analyses, due to unconfirmed immunisation status. The demographic characteristics of the study participants were generally comparable between the groups that had received one, two or three doses of vaccine six years previously. The unimmunised group were slightly older compared with girls from the one-dose group.

The proportion of girls who were seropositive for each HPV genotype six years after the last dose of 4vHPV, and also one month after the booster dose of 2vHPV, are shown in Table 1. Six years after the primary immunisation but prior to the booster dose, a significant difference was seen in the seropositivity rates for HPV31 in the girls who received one dose of 4vHPV when compared with three doses, but there were otherwise no significant difference in seropositivity rates between girls who received one or two doses of 4vHPV when compared with girls who received three doses. Amongst girls immunised six years previously with one to three doses of 4vHPV, and boosted with one dose of 2vHPV, no differences were seen in seropositivity rates to any tested HPV genotype, one month after the 2vHPV immunisation, whereas girls not previously immunised had significantly lower HPV seropositivity rates to all HPV genotypes. In girls who had six years previously received at least one dose of 4vHPV, seropositive rates for HPV31 (33–58%) were higher than for HPV33, 35, 52, and 58 (8–29%) and were also higher a month after a further dose of 2vHPV (95–99% for HPV31, and 54–93% for HPV 33, 35, 52, and 58).

**Figure 1:**
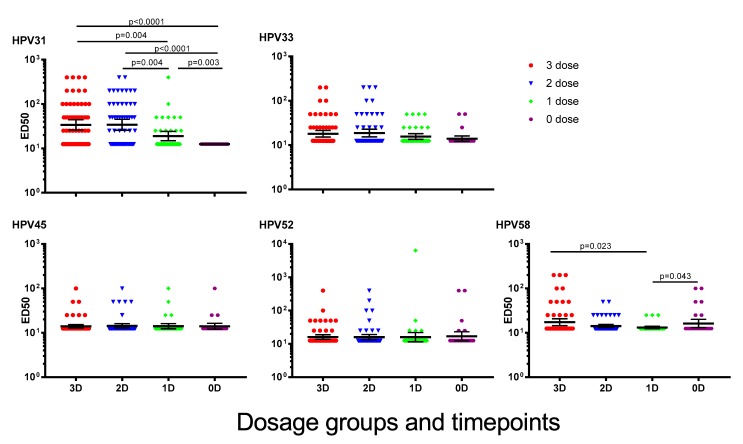
Cross-neutralising antibody (cross-NAb) titres to human papillomavirus (HPV) types 31, 33, 45, 52 and 58, six years after last dose of quadrivalent HPV vaccine (4vHPV). Data presented are geometric mean titre ±95% confidence interval. ED50 = effective dose 50.

Six years after immunisation with one, two or three doses of 4vHPV, GMTs for HPV31 were significantly higher than NAbs for HPV33, 45, 52 and 58 (Figure 1). Girls who received one dose of 4vHPV had significantly lower HPV31 and 58 NAb GMTs than girls who received three doses [HPV31, 3 dose: 33.90 (95% CI: 25.94–44.3), 1 dose: 18.95 (95% CI: 14.91–24.08), *p* = 0.004; HPV58, 3 dose: 17.31 (95% CI: 14.46–20.72), 1 dose: 13.17 (95% CI: 12.41–13.97), *p* = 0.023]. NAb GMTs to HPV31 for girls immunised six years previously were higher than for unimmunised girls [GMT: 12.5 (95% CI 12.5–12.5), *p* = 0.003]. Six girls previously unimmunised were seropositive for HPV58 at recruitment to the current study, and GMT for this HPV genotype were not significantly different between previously unimmunised girls and girls who previously received three doses of 4vHPV (3 dose: 17.31 (95% CI: 14.46–20.72), 0 dose: 16.21 (95% CI: 13.04–20.15, *p* = 0.661), and were marginally higher than in girls who had previously received one dose of 4vHPV (GMT: 13.17 (95%CI: 12.41–13.97, *p* = 0.043). No significant differences in the GMTs for HPV33, 45 and 52 were found between girls who had previously received three doses of 4vHPV and the other dosage groups.

**Figure 2:**
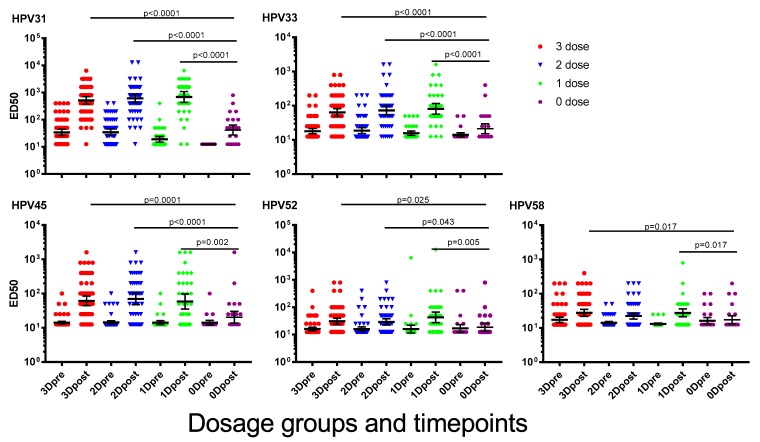
Cross-neutralising antibody (NAb) titres to human papillomavirus (HPV) types 31, 33, 45, 52 and 58, one month after a “booster” dose of bivalent HPV vaccine (2vHPV). Data presented are geometric mean titres ±95% confidence interval. ED50 = effective dose 50.

A booster immunisation with 2vHPV was given to all girls in the study to determine immunological memory responses to vaccine types HPV16 and 18 [22]. Here we report on the NAb responses to potentially cross-reactive types following the booster immunisation with 2vHPV. Following the 2vHPV immunisation, the NAb titres to HPV31, 33, 45, 52 and 58 increased significantly (*p* < 0.001 for all HPV types) in girls who had previously received at least one dose of 4vHPV. Interestingly, girls who received one dose of 4vHPV earlier increased between 2- and 46-fold to a level that was similar to the two-and three-dose groups (Figure 2). NAb levels in girls who had previously received at least one dose of 4vHPV were significantly higher than those not previously immunised, for all HPV types measured except HPV58. A dose of 2vHPV in previously unimmunised girls significantly increased NAb levels for HPV31 and 33 (HPV31, *p* < 0.0001; HPV33, *p* = 0.02), but not for HPV45, 52 and 58 (HPV52, *p* = 0.077; HPV52, *p* = 0.694; HPV58, *p* = 0.713). There were no significant differences for any of the HPV types before or after a dose of 2vHPV when comparing the girls who received two doses of 4vHPV more or less than six months apart (Figure S1).

We next determined whether there were differences between ethnic groups in NAb responses to the tested HPV types. Six years following 4vHPV immunisation, significantly higher NAb titres for HPV31 and 45 were found for FID girls compared with iTaukei girls who had received two- or three doses (Supplementary Table S1). Higher NAb titres for HPV31 and 45 were also observed in FID girls than in iTaukei girls following a booster dose of 2vHPV immunisation in girls who had received two- or three doses of 4vHPV, except for HPV31 in girls who had received two doses of 4vHPV. After the booster 2vHPV immunisation, higher HPV31 NAb titres were observed in FID girls than iTaukei girls who had previously received one dose of 4vHPV (*p* = 0.010), while in girls who were previously unimmunised, significantly higher HPV58 NAb titres were found in iTaukei girls (*p* = 0.026) when compared with FID girls.

**Figure 3:**
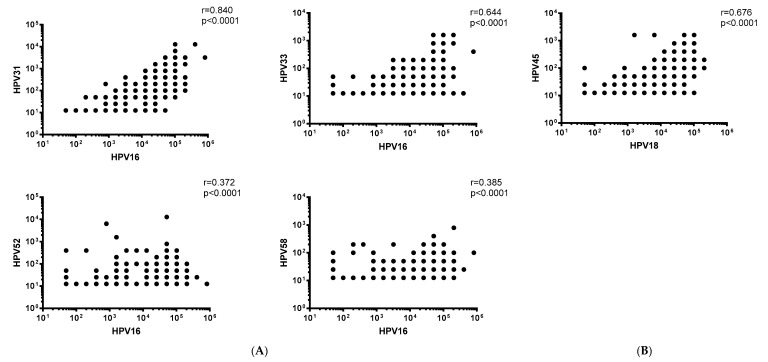
Scatterdot plots with correlations between (**A**) HPV16 and HPV31, 33, 52 and 58 NAb levels, as well as (**B**) HPV18 and 45 NAb levels. Data was pooled across all dosage groups as well as pre- and post-2vHPV. *r* = correlation coefficient.

We next undertook correlation analyses to determine the relationship between vaccine-type and non-vaccine type HPV NAb responses. Pooled analyses revealed moderate to strong positive correlations between HPV16 and HPV31 or HPV33 NAb levels (*r* = 0.84 and *r* = 0.644, respectively), as well as between HPV18 and HPV45 NAb levels (*r* = 0.676) (*p* < 0.0001) (Figure 3). The correlations were consistent when stratified by dosage group (Figure S2a–e). Weak, but significant positive correlations between HPV16 and HPV52 or 58 NAb levels were observed (*r* = 0.372 and *r* = 0.385, respectively) (*p* < 0.0001) (Figure 3), even when stratified by dosage groups (*p* < 0.01; except for the zero-dose group (HPV52, *p* = 0.148; HPV58, *p* = 0.555) (Figure S2a–e).

## 4. Discussion

To our knowledge, this is the longest follow up study to date documenting cross-NAb levels following HPV immunisation, and includes reduced-dose schedules. Our data suggests that two or one dose of 4vHPV may provide additional protection against HPV31 at least for six years. In addition, a broader immunological effect was observed post-2vHPV for all non-vaccine type measured in girls who received at least one dose of 4vHPV, although the clinical significance of this finding is unknown as there is currently no recognised level of NAb that is known to be protective.

Previous studies have reported cross-NAbs to HPV31 and 45, one month following two or three doses of 2vHPV or 4vHPV immunisation, at approximately 100-fold lower levels than vaccine-type NAb [16,17,18,19]. These cross-NAbs were found to persist for at least 5 and 2 years for 2vHPV and 4vHPV, respectively [16,17,25,26]. Our data extends these observations for HPV31 to six years (approximately 100-fold lower levels than vaccine-type NAb observed previously [22]), as well as to girls who received two doses of 4vHPV, suggesting that two doses given to girls under 15 years old may be sufficient to induce cross-protection to HPV31 in the long-term. The reason that only HPV31 NAb persisted but not the other types is an interesting finding and most likely due to its close relationship to HPV16 compared with other non-vaccine types [27]. Based on the literature, it is likely that cross-NAbs to HPV45 following 4vHPV were generated [16,17,18], and have waned below the assay detection limit after six years, although we are unable to confirm this as no data was collected six years prior. Studies evaluating NAbs to HPV33, 52 and 58 are limited, with only one study showing no significant increase in HPV52 and 58 cross-NAbs six months following the third dose of 2vHPV [19], making comparisons with our results difficult. The small number of previously unimmunised girls who were seropositive to HPV33, 45, 52 and 58 prior to a dose of 2vHPV suggests possible exposure to these types through natural infection and host immune responses. This could be due to their slightly older age compared with girls in the other groups. It is possible that some of the cross-NAb responses observed in the previously immunised groups may be due to current or previous exposure to the virus. However, it is unlikely that all the seroposivitve immunised girls are infected, and this is supported by an observational study in women in Fiji that found that the point prevalence of non-vaccine type detection (HPV31/33/45) was low (<8%) (F. Russell, unpublished data).

Vaccine efficacies (20–60%) and effectiveness (>60%) against HPV infection and cervical precancers caused by these HPV types (pooled analyses of HPV31/33/45 or HPV31/33/45/52/58) when given three doses of 2vHPV or 4vHPV have been demonstrated in clinical trials and population studies, respectively, with the highest efficacy observed for HPV31 [9,10,11,13,14,15,27]. For vaccine efficacy against HPV infection following two doses or even one dose of 2vHPV or 4vHPV in girls, similar cumulative incident HPV infections over seven years were observed when compared with three doses, although the numbers were small and the participants were not randomly selected [21,28]. In post-hoc analyses following one or two doses of 2vHPV given six months apart, a vaccine efficacy of 37% against incident HPV31/33/45 infection was also reported [11]. Ongoing trials evaluating single dose HPV vaccine schedule will provide important information on cross-protection for 2vHPV and 4vHPV [23]. The clinical significance of our data is unknown, but longitudinal studies evaluating both immunological and virological end points will be important in addressing cross-NAbs and cross-protection, particularly for reduced-dose schedules.

The booster responses to each HPV type 31, 33, 45, 52 and 58 following 2vHPV were similar across girls who previously received at least one dose of 4vHPV, and these were consistent with our earlier findings for HPV16/18 [22], suggesting immune memory to these cross-reactive types. Previous studies have shown HPV31- and HPV45-specific memory B cell responses following two- or three doses of 2vHPV or 4vHPV, which were still detectable in 50% of individuals after two years [16,17]. Our findings now show that HPV31 cross-NAb responses are detectable six years after 4vHPV and can be boosted by a later dose of 2vHPV. This is significant, particularly in LMICs currently using 4vHPV or 2vHPV and where the newly-licensed 9vHPV may be inaccessible, as broader protection against non-vaccine types could also occur. It is worth noting that girls who received an initial series of two or three doses are not recommended to be boosted routinely, rather that this was evaluated for the purpose of this study.

In previously unimmunised girls, HPV31 and 33 NAb titres, but not titres for HPV45, 52 and 58, increased significantly after 2vHPV, suggesting that one dose of 2vHPV may protect against HPV31 and 33 in the short term. These data are likely to be an underestimation since girls not previously immunised were greater than 15 years old when they received their first vaccine dose, and higher NAb responses are usually observed in younger girls under 15 years old [29,30,31]. Higher NAb to HPV31 and 45 are reported for 2vHPV than 4vHPV, which can be attributed to the AS04 adjuvant in 2vHPV [17,18,32]. A systematic review and meta-analysis also found higher vaccine efficacy estimates against HPV infections and lesions associated with HPV31, 33, and 45 for 2vHPV compared with 4vHPV, although the differences were not all significant [33].

Lower cross-NAb levels to HPV31 and HPV45 were observed for the iTaukei girls compared with FID girls in the two- and three-dose groups, consistent with our previous findings for HPV16 and HPV18 [22], although we did not power the study for these stratified analyses. The reasons for this difference is not known, although BMI may be one factor as iTaukei girls in our study had significantly higher BMI than FID girls [22]. Whether these findings have any clinical implications is not known, however continued surveillance of any vaccine failures is crucial, particularly for individuals or ethnic groups that induce lower immunity following HPV immunisation.

Levels of cross-NAb did not differ for any of the HPV types examined when the interval between the first and second dose was within or greater than six months. Higher immunogenicity or non-inferior antibody responses (when compared with a three-dose schedule) are usually observed for vaccine types when the second dose is given after more than six months [25,34,35]. This is also likely true for cross-NAbs, although there are only one other study showing similar HPV31 and 45 antibody responses between girls who received two doses of 2vHPV separated by six months and girls who received the standard three-dose schedule [35].

The major limitation of this study was the small sample size, particularly for the secondary analyses and the potential selection bias shown in the differences in participants’ age and education at enrolment as mentioned previously [22]. A strength of this study was the length of follow-up being one of the longest to date to report cross-NAb levels, as well as the inclusion of both one- and two-dose schedules, with data on all 5 HPV types that are included in 9vHPV. We also did not have any longitudinal HPV infection data, or information on participants’ sexual behaviours (i.e., whether they are sexually active and the number of sexual partners) in this cohort which may have influence the NAb responses, although as previously mentioned the prevalence for HPV31/33/45 in this population is low (F. Russell, unpublished data).

## 5. Conclusions

This study documented long-term cross-NAb response to HPV31 following reduced-dose 4vHPV vaccine schedules. Importantly, girls who were immunised with at least one dose of 4vHPV had HPV31 NAbs that persisted for six years and that a booster dose of 2vHPV enhanced cross-NAbs to all five non-vaccine types measured to a similar level as the two-and three-dose groups. How these findings relate to clinical protection is not known, but it is likely that 4vHPV will provide additional benefit by providing broader protection to HPV31, which is the 4th most common HPV type causing infections and invasive cervical cancer worldwide (4% in both cases) [36,37]. Further research into the long-term immunogenicity and efficacy of reduced-dose HPV schedules, using 2vHPV or 4vHPV, should be a continued priority to facilitate the introduction of HPV vaccine in LMICs to reduce the high burden of cervical cancer and HPV-associated disease.

## Figures and Tables

**Table 1:** Comparison of seropositivity rates to HPV 31, 33, 45, 52 and 58, six years following 4vHPV (Visit 1) and one-month following a dose of 2vHPV (Visit 2) between the three-dose group and zero-, one- or two-dose groups.

	Dosage Group	N	HPV Type									
			31		33		45		52		58	
			GMT (95% CI)	*p*-Value	GMT (95% CI)	*p*-Value	GMT (95% CI)	*p*-Value	GMT (95% CI)	*p*-Value	GMT (95% CI)	*p*-Value
**Visit 1**	3	66	38 (57.6%)	-	19 (28.8%)	-	7 (10.6%)	-	12 (18.0%)	-	14 (21.2%)	-
	2	60	33 (55.0%)	0.858	16 (26.7%)	0.844	6 (10.0%)	1.00	9 (15.0%)	0.811	9 (15.0%)	0.489
	1	40	13 (32.5%)	**0.016**	9 (22.5%)	0.506	4 (10.0%)	1.00	5 (12.5%)	0.587	3 (7.5%)	0.099
	0	32	0 (0%)	**<0.001**	3 (9.4%)	**0.039**	3 (9.4%)	1.00	5 (15.6%)	1.00	6 (20.7%)	1.00
**Visit 2**	3	66	65 (98.5%)	-	59 (88.1%)	-	52 (78.8%)	-	38 (57.6%)	-	36 (54.5%)	-
	2	59	58 (98.3%)	0.605	51 (86.4%)	0.594	41 (69.5%)	0.225	32 (54.2%)	0.720	39 (66.1%)	0.277
	1	40	38 (95.0%)	0.555	37 (92.5%)	0.739	26 (65%)	0.172	27 (69.2%)	0.411	28 (70.0%)	0.152
	0	30	22 (68.8%)	**<0.05**	10 (33.3%)	**<0.001**	9 (30%)	**<0.001**	6 (18.8%)	**<0.001**	6 (20.0%)	**0.001**

Bolded *p*-values represent statistically significant differences comparing the 3-dose group and 0-, 1- or 2-dose groups.

## Supplementary Materials

The following are available online at https://www.mdpi.com/2076-393X/7/4/200/s1, Figure S1: Cross-NAb titres to HPV31, 33, 45, 52 and 58 in 2-dose group, stratified by timing of the 2nd dose (<6 months, *n* = 22; ≥6 months, *n* = 38). Data presented are geometric mean titres ±95% confidence interval. ED50 = effective dose 50, Table S1: Geometric mean titres (GMTs) and 95% CI for HPV31, 33, 45, 52 and 58 for each dosage group, stratified by ethnicity, Figure S2: Correlation between HPV16 and HPV31 (**a**), 33 (**b**), 52 (**c**) and 58 (**d**), as well as HPV18 and 45 (**e**) NAb titres for all dosage groups. All data from both pre- and post-2vHPV are shown on the graphs. r = correlation coefficient.

## References

[1] Future I/II Study Group (2010). Four year efficacy of prophylactic human papillomavirus quadrivalent vaccine against low grade cervical, vulvar, and vaginal intraepithelial neoplasia and anogenital warts: Randomised controlled trial. BMJ.

[2] Future II Study Group (2007). Quadrivalent vaccine against human papillomavirus to prevent high-grade cervical lesions. N. Engl. J. Med..

[3] Lehtinen M., Paavonen J., Wheeler C.M., Jaisamrarn U., Garland S.M., Castellsagué X., Skinner S.R., Apter D., Naud P., Salmerón J. (2012). Overall efficacy of HPV-16/18 AS04-adjuvanted vaccine against grade 3 or greater cervical intraepithelial neoplasia: 4-year end-of-study analysis of the randomised, double-blind PATRICIA trial. Lancet Oncol..

[4] Paavonen J., Naud P., Salmerón J., Wheeler C.M., Chow S.N., Apter D., Kitchener H., Castellsague X., Teixeira J.C., Skinner S.R. (2009). Efficacy of human papillomavirus (HPV)-16/18 AS04-adjuvanted vaccine against cervical infection and precancer caused by oncogenic HPV types (PATRICIA): Final analysis of a double-blind, randomised study in young women. Lancet.

[5] Hildesheim A., Wacholder S., Catteau G., Struyf F., Dubin G., Herrero R., CVT Group (2014). Efficacy of the HPV-16/18 vaccine: Final according to protocol results from the blinded phase of the randomized Costa Rica HPV-16/18 vaccine trial. Vaccine.

[6] Artemchuk H., Eriksson T., Poljak M., Surcel H.M., Dillner J., Lehtinen M., Faust H. (2018). Long-Term Antibody Response to Human Papillomavirus Vaccines: Up to 12 Years Follow-Up in the Finnish Maternity Cohort. J. Infect. Dis..

[7] De Martel C., Plummer M., Vignat J., Franceschi S. (2017). Worldwide burden of cancer attributable to HPV by site, country and HPV type. Int. J. Cancer.

[8] De Villiers E.M., Fauquet C., Broker T.R., Bernard H.U., Zur Hausen H. (2004). Classification of papillomaviruses. Virology.

[9] Brown D.R., Kjaer S.K., Sigurdsson K., Iversen O.E., Hernandez-Avila M., Wheeler C.M., Perez G., Koutsky L.A., Tay E.H., Garcia P. (2009). The impact of quadrivalent human papillomavirus (HPV; types 6, 11, 16, and 18) L1 virus-like particle vaccine on infection and disease due to oncogenic nonvaccine HPV types in generally HPV-naive women aged 16–26 years. J. Infect. Dis..

[10] Wheeler C.M., Castellsagué X., Garland S.M., Szarewski A., Paavonen J., Naud P., Salmerón J., Chow S.N., Apter D., Kitchener H. (2012). Cross-protective efficacy of HPV-16/18 AS04-adjuvanted vaccine against cervical infection and precancer caused by non-vaccine oncogenic HPV types: 4-year end-of-study analysis of the randomised, double-blind PATRICIA trial. Lancet Oncol..

[11] Kreimer A.R., Struyf F., Del Rosario-Raymundo M.R., Hildesheim A., Skinner S.R., Wacholder S., Garland S.M., Herrero R., David M.P., Wheeler C.M. (2015). Efficacy of fewer than three doses of an HPV-16/18 AS04-adjuvanted vaccine: Combined analysis of data from the Costa Rica Vaccine and PATRICIA trials. Lancet Oncol..

[12] Ryser M., Berlaimont V., Karkada N., Mihalyi A., Rappuoli R., van der Most R. (2019). Post-hoc analysis from phase III trials of human papillomavirus vaccines: Considerations on impact on non-vaccine types. Expert Rev. Vaccin..

[13] Cameron R.L., Kavanagh K., Pan J., Love J., Cuschieri K., Robertson C., Ahmed S., Palmer T., Pollock K.G. (2016). Human Papillomavirus Prevalence and Herd Immunity after Introduction of Vaccination Program, Scotland, 2009–2013. Emerg. Infect Dis..

[14] Tabrizi S.N., Brotherton J.M., Kaldor J.M., Skinner S.R., Liu B., Bateson D., McNamee K., Garefalakis M., Phillips S., Cummins E. (2014). Assessment of herd immunity and cross-protection after a human papillomavirus vaccination programme in Australia: A repeat cross-sectional study. Lancet Infect. Dis..

[15] Donken R., King A.J., Bogaards J.A., Woestenberg P.J., Meijer C.J.L.M., de Melker H.E. (2018). High effectiveness of the bivalent HPV vaccine up to six years post-vaccination against incident and persistent HPV infections in young Dutch females. J. Infect. Dis..

[16] Einstein M.H., Baron M., Levin M.J., Chatterjee A., Fox B., Scholar S., Rosen J., Chakhtoura N., Lebacq M., van der Most R. (2011). Comparison of the immunogenicity of the human papillomavirus (HPV)-16/18 vaccine and the HPV-6/11/16/18 vaccine for oncogenic non-vaccine types HPV-31 and HPV-45 in healthy women aged 18–45 years. Hum. Vaccin..

[17] Godi A., Bissett S.L., Miller E., Beddows S. (2015). Relationship between Humoral Immune Responses against HPV16, HPV18, HPV31 and HPV45 in 12-15 Year Old Girls Receiving Cervarix(R) or Gardasil(R) Vaccine. PLoS ONE.

[18] Barzon L., Squarzon L., Masiero S., Pacenti M., Marcati G., Mantelli B., Gabrielli L., Pascucci M.G., Lazzarotto T., Caputo A. (2014). Neutralizing and cross-neutralizing antibody titres induced by bivalent and quadrivalent human papillomavirus vaccines in the target population of organized vaccination programmes. Vaccine.

[19] Kemp T.J., Hildesheim A., Safaeian M., Dauner J.G., Pan Y., Porras C., Schiller J.T., Lowy D.R., Herrero R., Pinto L.A. (2011). HPV16/18 L1 VLP vaccine induces cross-neutralizing antibodies that may mediate cross-protection. Vaccine.

[20] World Health Organization (2014). Meeting of the Strategic Advisory Group of Experts on immunization, April 2014—conclusions and recommendations. Wkly. Epidemiol. Rec..

[21] Sankaranarayanan R., Joshi S., Muwonge R., Esmy P.O., Basu P., Prabhu P., Bhatla N., Nene B.M., Shaw J., Poli U.R.R. (2018). Can a single dose of human papillomavirus (HPV) vaccine prevent cervical cancer? Early findings from an Indian study. Vaccine.

[22] Toh Z.Q., Russell F.M., Reyburn R., Fong J., Tuivaga E., Ratu T., Nguyen C.D., Devi R., Kama M., Matanitobua S. (2017). Sustained Antibody Responses six years Following 1, 2, or three doses of Quadrivalent Human Papillomavirus (HPV) Vaccine in Adolescent Fijian Girls, and Subsequent Responses to a Single Dose of Bivalent HPV Vaccine: A Prospective Cohort Study. Clin. Infect. Dis..

[23] Kreimer A.R., Herrero R., Sampson J.N., Porras C., Lowy D.R., Schiller J.T., Schiffman M., Rodriguez A.C., Chanock S., Jimenez S. (2018). Evidence for single-dose protection by the bivalent HPV vaccine-Review of the Costa Rica HPV vaccine trial and future research studies. Vaccine.

[24] Toh Z.Q., Cheow K.W.B., Russell F.M., Hoe E., Reyburn R., Fong J., Tuivaga E., Ratu F.T., Nguyen C.D., Matanitobua S. (2018). Cellular Immune Responses six years Following 1, 2, or three doses of Quadrivalent HPV Vaccine in Fijian Girls and Subsequent Responses to a Dose of Bivalent HPV Vaccine. Open Forum Infect. Dis..

[25] Romanowski B., Schwarz T.F., Ferguson L.M., Ferguson M., Peters K., Dionne M., Schulze K., Ramjattan B., Hillemanns P., Behre U. (2014). Immune response to the HPV-16/18 AS04-adjuvanted vaccine administered as a 2-dose or 3-dose schedule up to 4 years after vaccination: Results from a randomized study. Hum. Vaccin. Immunother..

[26] Folschweiller N., Behre U., Dionne M., Durando P., Esposito S., Ferguson L., Ferguson M., Hillemanns P., McNeil S.A., Peters K. (2019). Long-term Cross-reactivity Against Nonvaccine Human Papillomavirus Types 31 and 45 After 2-or 3-Dose Schedules of the AS04-Adjuvanted Human HPV-16/18 Vaccine. J. Infect. Dis..

[27] Bogaards J.A., van der Weele P., Woestenberg P.J., van Benthem B.H.B., King J.A. (2019). Bivalent Human Papillomavirus (HPV) Vaccine Effectiveness Correlates with Phylogenetic Distance From HPV Vaccine Types 16 and 18. J. Infect. Dis..

[28] Safaeian M., Sampson J.N., Pan Y., Porras C., Kemp T.J., Herrero R., Quint W., van Doorn L.J., Schussler J., Lowy D.R. (2018). Durability of Protection Afforded by Fewer Doses of the HPV16/18 Vaccine: The CVT Trial. J. Natl. Cancer Inst..

[29] Block S.L., Nolan T., Sattler C., Barr E., Giacoletti K.E., Marchant C.D., Castellsagué X., Rusche S.A., Lukac S., Bryan J.T. (2006). Comparison of the immunogenicity and reactogenicity of a prophylactic quadrivalent human papillomavirus (types 6, 11, 16, and 18) L1 virus-like particle vaccine in male and female adolescents and young adult women. Pediatrics.

[30] Pedersen C., Petaja T., Strauss G., Rumke H.C., Poder A., Richardus J.H., Spiessens B., Descamps D., Hardt K., Lehtinen M. (2007). Immunization of early adolescent females with human papillomavirus type 16 and 18 L1 virus-like particle vaccine containing AS04 adjuvant. J. Adolesc. Health.

[31] Petaja T., Keränen H., Karppa T., Kawa A., Lantela S., Siitari-Mattila M., Levänen H., Tocklin T., Godeaux O., Lehtinen M. (2009). Immunogenicity and safety of human papillomavirus (HPV)-16/18 AS04-adjuvanted vaccine in healthy boys aged 10–18 years. J. Adolesc. Health.

[32] Garcon N., Wettendorff M., Van Mechelen M. (2011). Role of AS04 in human papillomavirus vaccine: Mode of action and clinical profile. Expert Opin. Biol. Ther..

[33] Malagon T., Drolet M., Boily M.C., Franco E.L., Jit M., Brisson J., Brisson M. (2012). Cross-protective efficacy of two human papillomavirus vaccines: A systematic review and meta-analysis. Lancet Infect. Dis..

[34] Sankaranarayanan R., Prabhu P.R., Pawlita M., Gheit T., Bhatla N., Muwonge R., Nene B.M., Esmy P.O., Joshi S., Poli U.R. (2016). Immunogenicity and HPV infection after one, two, and three doses of quadrivalent HPV vaccine in girls in India: A multicentre prospective cohort study. Lancet Oncol..

[35] Puthanakit T., Huang L.M., Chiu C.H., Tang R.B., Schwarz T.F., Esposito S., Frenette L., Giaquinto C., McNeil S., Rheault P. (2016). Randomized Open Trial Comparing 2-Dose Regimens of the Human Papillomavirus 16/18 AS04-Adjuvanted Vaccine in Girls Aged 9–14 Years Versus a 3-Dose Regimen in Women Aged 15–25 Years. J. Infect. Dis..

[36] Bruni L., Diaz M., Castellsagué X., Ferrer E., Bosch F.X., de Sanjosé S. (2010). Cervical human papillomavirus prevalence in 5 continents: Meta-analysis of 1 million women with normal cytological findings. J. Infect. Dis..

[37] De Sanjose S., Diaz M., Castellsagué X., Clifford G., Bruni L., Muñoz N., Bosch F.X. (2007). Worldwide prevalence and genotype distribution of cervical human papillomavirus DNA in women with normal cytology: A meta-analysis. Lancet Infect. Dis..

