# Supplementary material for: Selective Persistence of HPV Cross-Neutralising Antibodies following Reduced-Dose HPV Vaccine Schedules
Source: Vaccines (Basel). 2019 Nov 28;7(4):200. doi: 10.3390/vaccines7040200 (PMC6963583; doi:10.3390/vaccines7040200)
Supplement: Supplementary file 1 [file vaccines-07-00200-s001.pdf]

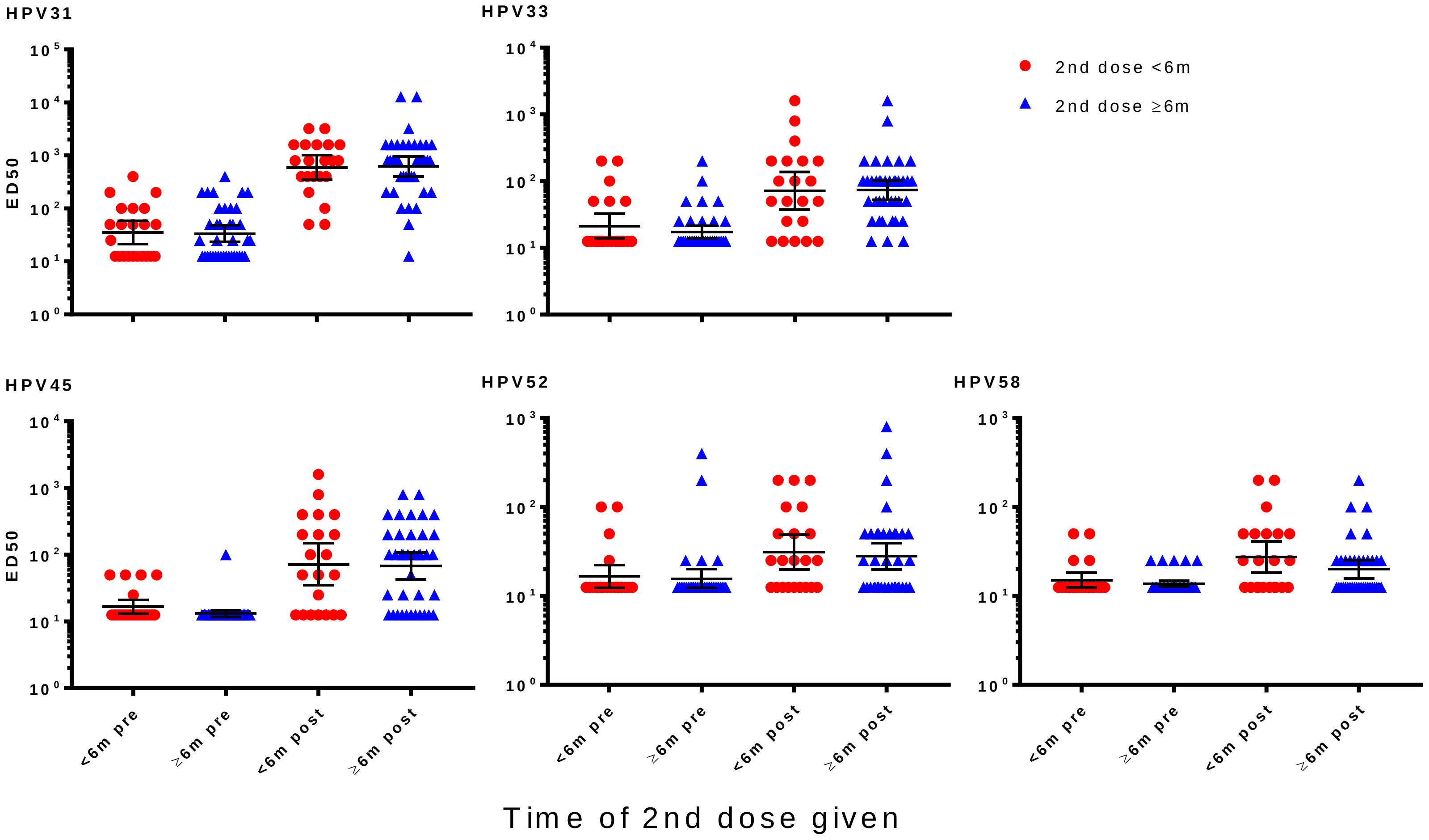

Figure S1. Cross-NAb titers to HPV31, 33, 45, 52 and 58 in 2-dose group, stratified by timing of the 2nd dose (<6 months, n=22; ≥6 months, n=38). Data presented are geometric mean titers ± 95% confidence interval. ED50 = effective dose 50.

Table S1. Geometric mean titres (GMTs) and 95% CI for HPV31, 33, 45, 52 and 58 for each dosage group, stratified by ethnicity.

Time point	Dosage group	Ethnic group	N	HPV type									
				31		33		45		52		58	
				GMT (95%CI)	p	GMT (95%CI)	p	GMT (95%CI)	p	GMT (95%CI)	p	GMT (95%CI)	p
Visit 1	3	i-Taukei	36	26.5 (19, 36)		18.3 (14.4, 23.4)		12.7 (12, 13)		16.7 (14, 20)		18.4 (14, 24)	
		FID	30	45.6 (29, 71)	0.043	17.7 (13.7, 22.7)	0.823	15.8 (13, 19)	0.022	15.4 (12, 20)	0.606	16 (13, 21)	0.474
	2	i-Taukei	32	25.6 (19, 35)		20.6 (14.8, 28.7)		13.1 (12, 14)		15.5 (12, 20)		14.6 (13, 17)	
		FID	28	47.6 (30, 77)	0.028	16.8 (13.4, 21.1)	0.323	16 (13, 20)	0.072	16.4 912, 22)	0.760	13.8 (13, 15)	0.536
	1	i-Taukei	17	21.2 (13, 36)		18.0 (13.6, 24.0)		15.3 (12, 20)		22.1 (10, 48)		13.6 (12, 15)	
		FID	23	17.4 (14, 22)	0.415	14.1 (12.2, 16.3)	0.090	13.3 (12, 15)	0.282	12.5	0.077	12.9 (12, 14)	0.392
	0	i-Taukei	15	12.5		15.8 (12, 21)		15.0 (11, 20)		17.3 (10, 28)		20.8 (13, 32)	
		FID	17	12.5	-	12.5	0.067	13.0 (12, 14)	0.315	16.6 (11, 26)	0.905	13.0 (12, 14)	0.026
Visit 2	3	i-Taukei	36	385 (244, 607)		58.3 (42.5, 80.1)		38.9 (27, 56)		24.5 (18, 33)		29.2 (21, 31)	
		FID	30	729 (473, 1126)	0.045	70.7 (45.8, 109)	0.459	105 (61, 179)	0.002	40.6 (26, 64)	0.05	26.2 (19, 37)	0.648
	2	i-Taukei	31	598 (398, 900)		83.6 (54.7, 128)		41.8 (26, 67)		28.6 (19, 42)		25.0 (18, 34)	
		FID	28	625 (352, 1107)	0.899	62.5 (39.3, 99.3)	0.346	122 (70, 214)	0.004	29.7 (20, 44)	0.885	20.0 (15, 27)	0.284
	1	i-Taukei	17	354 (146, 857)		81.6 (40.6, 164)		75.2 (28, 200)		39.2 (17, 92)		28.3 (17, 47)	
		FID	23	1114 (745, 1667)	0.010	81.0 (53.8, 122)	0.985	48.5 (27, 87)	0.398	41.4 (26, 67)	0.687	27.4 (20, 38)	0.907
	0	i-Taukei	15	37.9 (20, 72)		23.9 (14, 41)		17.3 (12,26)		21.8 (11, 42)		21.8 (13, 38)	
		FID	15	45.6 (25, 84)	0.660	19.0 (12, 30)	0.494	23.9 (12,49)	0.408	14.5 (12, 18)	0.838	13.7 (12, 16)	0.089

p, p-value. Bolded p-values represent statistically significant differences between the ethnic groups.

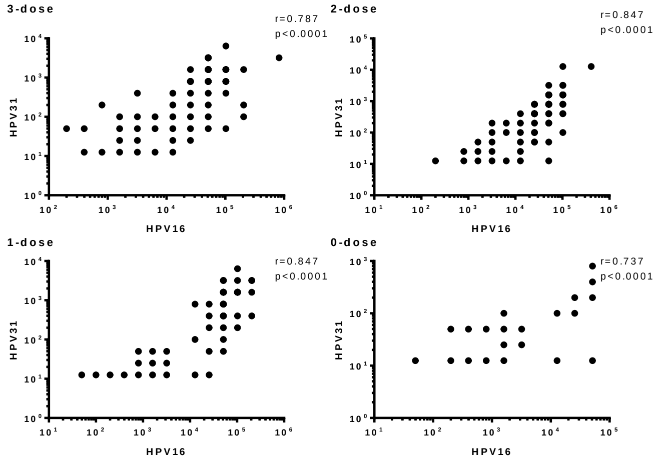
3-dose
r=0.787
p<0.0001
2-dose
r=0.847
p<0.0001
1-dose
r=0.847
p<0.0001
0-dose
r=0.737
p<0.0001
HPV31
HPV16

(**a**)

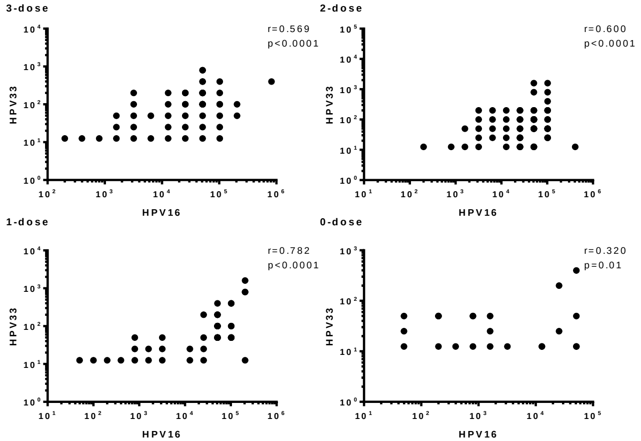
3-dose
r=0.569
p<0.0001
2-dose
r=0.600
p<0.0001
1-dose
r=0.782
p<0.0001
0-dose
r=0.320
p=0.01
HPV33
HPV16

(**b**)

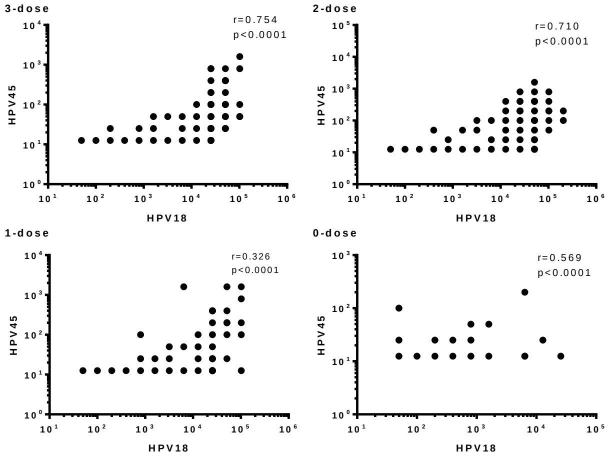
3-dose
r=0.754
p<0.0001
2-dose
r=0.710
p<0.0001
1-dose
r=0.326
p<0.0001
0-dose
r=0.569
p<0.0001
HPV45
HPV18

(**c**)

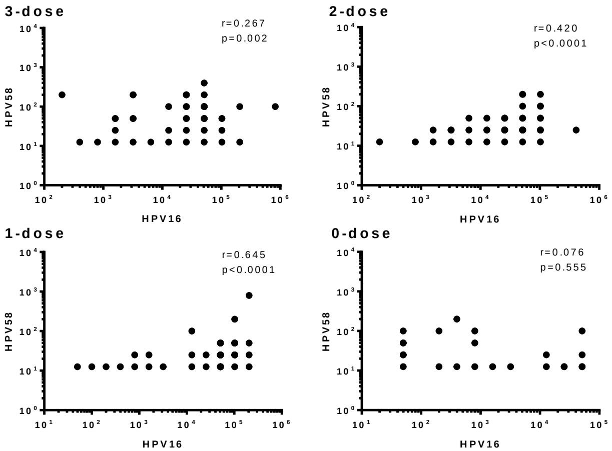
3-dose
r=0.267
p=0.002
2-dose
r=0.420
p<0.0001
1-dose
r=0.645
p<0.0001
0-dose
r=0.076
p=0.555
HPV58
HPV16

(**d**)

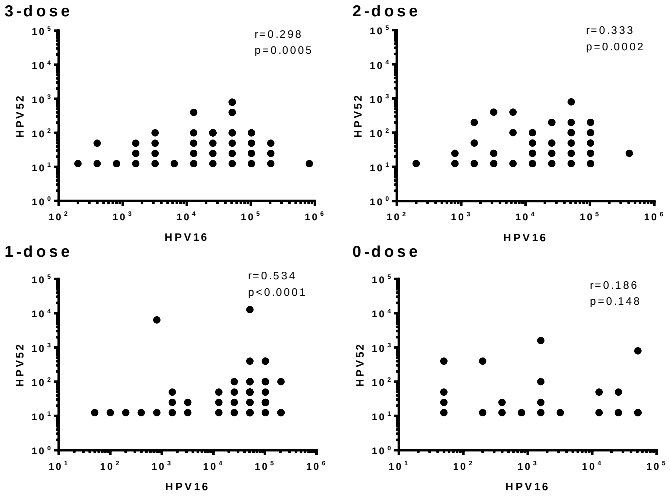

(**e**)

Figure S2. Correlation between HPV16 and HPV31 (**a**), 33 (**b**), 52 (**c**) and 58 (**d**), as well as HPV18 and 45 (**e**) NAb titres for all dosage groups. All data from both pre- and post-2vHPV are shown on the graphs. *r* = correlation coefficient.
